# Pharmacogenomic Profiling of ADME Gene Variants: Current Challenges and Validation Perspectives

**DOI:** 10.3390/ht7040040

**Published:** 2018-12-18

**Authors:** Mariamena Arbitrio, Maria Teresa Di Martino, Francesca Scionti, Vito Barbieri, Licia Pensabene, Pierosandro Tagliaferri

**Affiliations:** 1 Institute of Neurological Sciences, UOS of Pharmacology, 88100 Catanzaro, Italy; mariamena.arbitrio@cnr.it; 2 Department of Experimental and Clinical Medicine, Magna Graecia University, Salvatore Venuta University Campus, 88100 Catanzaro, Italy; teresadm@unicz.it (M.T.D.M.); scionti@unicz.it (F.S.); 3 Medical Oncology Unit, Mater Domini Hospital, Salvatore Venuta University Campus, 88100 Catanzaro, Italy; barbieri@unicz.it; 4 Department of Medical and Surgical Sciences Pediatric Unit, Magna Graecia University, 88100 Catanzaro, Italy; pensabene@unicz.it

**Keywords:** pharmacogenomics, ADME genes, cancer, single nucleotide polymorphisms

## Abstract

In the past decades, many efforts have been made to individualize medical treatments, taking into account molecular profiles and the individual genetic background. The development of molecularly targeted drugs and immunotherapy have revolutionized medical treatments but the inter-patient variability in the anti-tumor drug pharmacokinetics (PK) and pharmacodynamics can be explained, at least in part, by genetic variations in genes encoding drug metabolizing enzymes and transporters (ADME) or in genes encoding drug receptors. Here, we focus on high-throughput technologies applied for PK screening for the identification of predictive biomarkers of efficacy or toxicity in cancer treatment, whose application in clinical practice could promote personalized treatments tailored on individual’s genetic make-up. Pharmacogenomic tools have been implemented and the clinical utility of pharmacogenetic screening could increase safety in patients for the identification of drug metabolism-related biomarkers for a personalized medicine. Although pharmacogenomic studies were performed in adult cohorts, pharmacogenetic pediatric research has yielded promising results. Additionally, we discuss the current challenges and theoretical bases for the implementation of pharmacogenetic tests for translation in the clinical practice taking into account that pharmacogenomics platforms are discovery oriented and must open the way for the setting of robust tests suitable for daily practice.

## 1. Introduction

Over the past few decades, pharmacogenomics (PGx) studies contributed to the understanding of the interindividual variability in response to drug treatment opening a new scenario toward personalized approaches in the modern health care. After the completion of the Human Genome Project (HGP) in 2001, the knowledge of human sequence variation allowed advances in targeted drug discovery and in the prediction of drug sensitivity. The deeper understanding of human genetic findings and interindividual variations in drug response has recently disclosed new opportunities for personalized treatments. DNA research has moved from human genome sequencing to the mapping of genetic variations for investigating differences among individuals.

The HapMap Project brought to identify haplotype blocks of common single nucleotide polymorphisms (SNPs) in different human populations which were co-inherited and in linkage disequilibrium (LD) with specific genetic variants with important functional variability [1].

The evolution of high-throughput technologies allowed the identification of new genomic correlates to human disease, drug response, and adverse reactions, with major benefits for patient management. In particular, the integration of information deriving from multidisciplinary approaches to disease management allowed a tailored strategy that overcame the empirical “one fits all” treatment paradigm. The genetic research implemented the study of both relatively rare monogenic diseases and common and genetically complex diseases, such as cancer. A major aim was to clarify the genetic basis of variable drug responses and adverse drug reactions (ADRs).

The study of genetically influenced variations in drug response and ADRs risk is known as PGx which, in particular, focuses on gene-drug relationships highlighting the role of genetic, biological, and molecular mechanisms. Polymorphic variants in genes related to drug absorption, distribution, metabolism and excretion (ADME) contribute to interindividual variability in drug efficacy and adverse effects. Hence, the identification of PGx biomarkers has the potentiality of optimizing treatment for individuals toward the precision medicine paradigm. In this context, the major goal for cancer treatment in a personalized approach is to select the right dose of the right drug for a right tumor in the right patient. The interindividual variability in drug efficacy and toxicity can be solved by knowledge of somatic (tumor) or germline (patients) variations. The somatic non-inherited mutations in tumor tissue can define disease subtypes and predict drug efficacy and clinical outcome of different tumors [2]. The germline genome can impact treatment outcome in terms of pharmacokinetics (PK) and pharmacodynamics (PD). Specifically, the activity of polymorphic variants in PK relevant ADME genes, coding for enzymes, transporters, cell membrane, and/or intracellular receptors or components of ion channels, can affect patient’s drug exposure and the efficacy and toxicity for many drugs, especially anti-tumor agents. The effects in PK changes become relevant in patients where genetic polymorphisms can alter the effect on the body and on drug metabolism [3]. The most common germline variations studied in PGx are SNPs, insertions, deletions, and copy number variations (CNVs). In the human genome, SNPs are common inherited variations (>1%) among people in which the DNA sequence may show variants at a single nucleotide level and which can have functional relevance when occurs in coding sequences or in regulatory regions. SNPs are inherited in haplotype blocks spanning within two high hyper-recombination sites (Hot Spot) and in strong LD with specific polymorphic variants in genes located in the same block. SNPs in strong LD can be used as markers of a specific haplotype [4,5]. Consequently, a modest number of common SNPs, selected from each block and considered as tag or tagging SNP (tSNP), would allow to identify the gene variants related to a disease or to interindividual variability in drug response or toxicity [6]. The approach based on tSNPs allowed to shift from a candidate-gene based approach to the genome-wide association study (GWAS). Further technologic tools, like germline SNP panel, can represent the ideal compromise between above mentioned genotyping methods. The purpose of this review is to critically discuss advantages and weaknesses of different PGx approaches for the discovery of germline predictive biomarkers in ADME genes comparing also potentialities and limitations of different PGx tools for pre-treatment testing for translation into common practice for personalized treatment.

## 2. Pharmacogenomics Approaches for Germline SNP Identification

Over the last years, more and more advanced and sophisticated technologies allowed the identification of genomic loci associated to interindividual variability in drug response, although the primary objective of these approaches was towards the mapping and discovery of genes that may have a role in disease development or risk [7]. GWAS are performed on large populations of unrelated individuals for simultaneous evaluation of SNPs and CNVs spanning the entire genome, in order to discover potential biomarkers in genes without validated evidence of function. It represents a powerful approach to identify genomic regions in which genetic variants in LD might confer risk for disease. GWAS can identify only common variants while the identification of rare variants is not allowed. During last years, the GWAS-based research identified several associations between specific chromosomal loci and different types of cancer [8]. However, this kind of study requires a huge number of different tests with stringent statistical correction which need very large samples [9]. In a subset of disease common and low-frequency (Minor Allele Frequency (MAF) > 0.5% and <5%) genetic variants can have small effects and can be identified by GWAS and/or imputation. On these loci the calculation of polygenic risk score, as sum of the number of risk alleles carried by an individual, could increase statistical power to find associations, and summarize the total genetic effect. Polygenic risk estimation combined with clinical risk factors can be useful to drive clinical or personal decision-making [10,11,12]. Differently, the approach widely used to study genetic susceptibility to complex diseases, including cancer, is still on the candidate gene paradigm which interrogates a single or small group of identified SNPs, whose role is recognized for a specific phenotype and can be used also for marker validation. Limitations on this type of studies is the hard replication of results, large number of false positives, small sample sizes, and limits to include all biologically relevant genes and polymorphisms [13]. Several PGx analyses have been conducted applying both approaches; however, for their statistical limitations, could not be considered as an ideal approach for PGx discovery. In fact, candidate-SNP studies showed high rate of false-positive findings and overestimation of effect sizes, whereas in GWAS correction for multiple comparisons are required to avoid false negatives. Therefore, either approaches have also restricted information to genes related to PD of different classes of drugs. Although these approaches are complementary for the identification of genetic contributions to common diseases and PGx studies, a valid compromise for the identification of polymorphic variants in genes related to the risk of ADRs is represented by pre-defined SNPs panel approach (Table 1). In fact, this approach leads to efficient simultaneous genotyping of candidate SNPs with known relevance while limiting the need of statistical stringency for multiple comparisons. SNP panels interrogate genetic variants in PK relevant genes but include also some SNPs in known PD relevant genes allowing the discovery and integration of known germline PK-PD genetic associations. 

## 3. Pharmacogenomics Tools Currently Available

In addition to PGx biomarkers used in clinical settings, there are numerous commercially available PGx-discovery tools on the market utilizing different genetic panels with different technology, coverage and performance, until now used for research only. Most are targeted genotyping assays in which probes are specifically designed on variants with well-defined drug-gene interactions and selected from the ADME gene list, created by Pharma-ADME. These high-throughput technologies spans from real-time multiplexed PCR-based methods using TaqMan probe chemistry (TaqMan Open Array PGx Express Panel (Thermo Fisher Scientific, Waltam, MA, USA)), microarrays (DMET and PharmacoScan, Thermo Fisher Scientific) to mass spectroscopy arrays (iPLEX^®^ ADME Pharmacogenetic Panel (Sequenom, San Diego, CA, USA)) (Table 2). 

Among PGx ADME SNPs panel, DMET^TM^ Plus array (Thermo Fisher Scientific) was the first available to our knowledge. Through the Targeted Genotyping System, the combination of molecular inversion probe (MIP) and single multiplexed assay based on quantitative real time-polymerase chain reaction (qPCR) allowed the analyses of several thousand SNPs in a single assay [14,15]. Starting from 1 μg of genomic DNA samples extracted from peripheral blood or saliva the DMET^TM^ assay tests 1936 (1931 SNPs and five CNVs) markers in 231 ADME genes whose role in drugs metabolism and elimination is recognized by Food and Drug Administration (FDA, Silver Spring, MD, USA) [16]. By DMET^TM^ platform it is possible to capture several markers, including copy-number variations, insertions/deletions, bi-allelic and tri-allelic SNPs [17]. The profiles for the genotyping call rates and concordance comparisons are generated by the DMET^®^ Console software (Thermo Fisher Scientific) which is based on the BRLMM (Bayesian Robust Linear Model with Mahalanobis distance classifier) algorithm. Predefined fixed or dynamic genotype boundaries are used, for comparison of each individual marker’s data, as the algorithms for all genotyping configurations. 

The reproducibility of genotyping results accuracy with Sanger sequencing is of 100% [17]. The tabular data provided by DMET^®^ Console can be analyzed by DMET-Analyzer by Fisher’s exact test which gives the data in a format easily readable and compatible for the statistical analysis [18]. The biological interpretation is the last step for each analyzed SNP, to achieve additional information stored in the Pharmacogenomics Knowledge Base (PharmGKB), in other external database of SNPs or in the critical evaluation of scientific literature in the case of SNPs of unknown role. DMET^TM^ platform was also used to investigate the inter-individual variability in common and uncommon diseases [17,18,19,20,21,22,23]. An example of clinical applicability of pre-emptive genotyping by DMET^TM^ platform array supplemented with a *CYP2D6* copy number assay was the PG4KDS protocol, St. Jude Children’s Research Hospital (Memphis, TN, USA) by which it was possible to provide in 1559 pediatric patients with different diseases a stepwise process for implementing gene/drug correlation, organizing data and obtaining consent from patients and families. In this scenario DMET^TM^ microarray platform has proven as efficient PGx tool for discovering new potential biomarkers influencing chemotherapy-induced toxicity or to identify the metabolizing phenotypes toward a translational perspective in clinical practice [24].

Despite no longer available on the market, the VeraCode ADME Core Panel (Illumina, Inc. San Diego, CA, USA) is another genotyping assay extensively used in PGx studies. This panel targets 184 variants in 34 genes involved in ADME genes [25]. This assay was applied for the PGx implementation project named Pharmacogenomic Resource for Enhanced Decisions in Care and Treatment (PREDICT) at Vanderbilt University Medical Center (VUMC) which focused first on antiplatelet therapy following placement of cardiovascular stents and its potential generalizability [26]. Approximately 3000 patients, most scheduled for cardiac catheterization, were enrolled and genotyped in one year, including *CYP2C19* variants that modulate response to clopidogrel. PGx sequencing tool utilizing targeted or whole-exome sequencing (WES) and whole-genome sequencing (WGS) have the advantage to discover rare alleles, poorly tagged by existing genotyping platforms, that may have important effects on drug response. An example of targeted sequencing is the Ion AmpliSeq PGx panel, a single pool of primers used to perform multiplex PCR for preparation of amplicon libraries from genomic hot spot regions that play critical role in ADME genes. However, despite the opportunity offered by next generation sequencing-based technologies to massively scan the human genome, clinical implementation of PGx testing is slow due to high costs and expertise required for molecular and data analysis and interpretation.

## 4. Candidate Biomarkers Discovery Process

The possibility to choose different high-throughput discovery driven platforms can improve the identification of intrinsic and extrinsic determinants related to interindividual variability in drugs exposure. In particular, drug efficacy and safety are influenced by extrinsic factors correlated to polytherapy’s (drug/drug interactions), while drug dose could be adjusted in consideration of genotype/phenotype correlation due to intrinsic factors [27]. Germline ADME genotyping has been performed in a wide range of therapeutic areas and diseases and has allowed pharmaceutical companies, academic scientists and regulatory agencies to investigate and achieve information on developmental compounds and marketed drugs, taking in consideration genetic polymorphisms in enzyme proteins and transporters. Extensive PGx research results improved drugs and patients ‘outcomes’. The more and more available PGx results will increase reliance on PGx testing into clinical practice. The advent of more comprehensive genetic testing allowed clinical discovery to expand the simultaneous study of a panel of genes with agnostic identification and a more attractive and cost-effective approach. 

**Figure 1:**
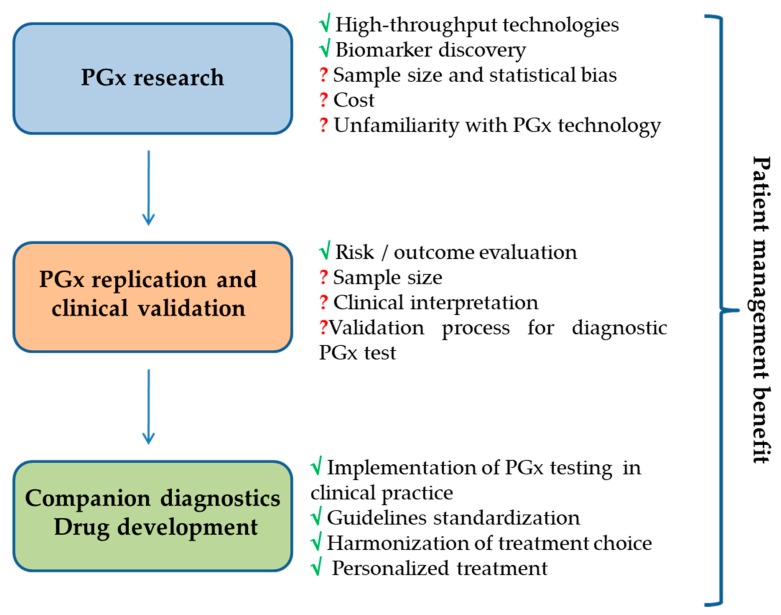
PGx biomarkers discovery process.

At this aim a crucial role is played by the pros and contras of discovery platforms, whose effectiveness is fundamental for wide spread PGx translation in the clinical practice (Figure 1). Until today, even if currently available tools explore similar SNP panels, technologic approach and specifications are different as well as test specificity and sensitivity related to genetic factors and the validation for diagnostic finality. In fact, presently many tests and kits are for research use only. The majority of PGx potential biomarkers are discovered in small sample sets and need statistical validation in large, well-defined external cohorts. The empowering of clinical research, as validation studies of discovered genetic variants, will help the optimization of sample size and the translation would finally lead to the assessment of clinical utility for identified drug–gene interactions in order to set robust biomarkers for clinical usage. Another issue to consider in targeted genotyping is the ethnic differences in the population, which might limit the selection of genomic variants common in specific population and missing in others [28]. An example is represented by CYP2C9*2 and CYP2C9*3, two variant alleles with reduced function important for response to warfarin which are common in Caucasians but rare in African-Americans [29]. Therefore, the choice of an appropriate assay might to be influenced by the target population, besides the assay cost and the validation for diagnostic biomarker test to perform in certified laboratory. The availability of PGx tests and knowledge of how to access them is important for successful implementation into clinical practice. The integration of data reported by databases with PGx information such as PharmGKB [30], the Clinical Pharmacogenetics Implementation Consortium (CPIC) [31] or the Gene SNPs database [32] and algorithms incorporating all information, could define prescribing guidelines associated to affected drugs with interpretation of results that could be translated in customized genetic laboratory test. 

## 5. Candidate Biomarkers Validation Process

The PGx derived biomarkers are becoming basic tools for precision medicine, theoretically appreciated by physicians and patients, whose introduction into daily clinical practice could help to contain the health costs of patient management. To introduce a biomarker into clinical practice is however necessary to follow a formal validation process based on regulatory agencies guidelines. In fact, all PGx discovery platforms we have discussed here, have an intrinsic risk of over-fittings, which may preclude confirmation outside the learning set. The results confirmation need to be validated through an alternative reference methods in order to avoid the risk of false positive and/or doubtful genotype results. Therefore, analytical validity is a critical step to assess genomic biomarker in replica studies in a separate and matched cohort for further clinical validation. The development of biomarker-based tests for clinical studies and clinical practice requires its reproducibility and scientific validation. At this aim, classical cross validation or a training set approaches can reduce the risk of over-fitting and are strictly required to gather strong evidence before progressing to validation in independent patient series and eventually incorporate the biomarker into a clinical test validated for a specific clinical use.

However, to assess the validity of any test, the “gold standard” is the confirmation of all computational procedures and candidate omics-based tests on independent sample set. The result of the process is an omics-discovered candidate biomarker that will proceed in test validation phase to assess analytical and clinical/biological validity. In all phases of development of a PGx test, statistics and bioinformatics validation are needed for further evaluation of clinical use and utility [33]. The test analytical validation requires severe qualitative or quantitative parameters as, for example, accuracy, reproducibility, linearity, reportable range, analytical sensitivity and specificity, and limit of detection [34]. For example, genomic analyses, as microarray or NGS, needs validation in orthogonal (sequencing) approaches. Biologic samples comparable to the patient specimens, on which the test will be used, are utilized for analytical validation and evaluated for clinical/biological validity. If clinical specimens are not available, alternative biological specimens (cell lines and test in vitro) can be used even if the variability evaluable with the clinical specimens it not seen in the biological specimens. In the validation phase, the operator who is performing and interpreting test results should not know the expected or previously achieved test results, in order to avoid the risk of bias and improve the likelihood of a successful translation in clinical trial and in common practice. 

Clinical/biological validation of a genomic biomarker includes also the confirmation of association with a functional phenotype important for clinical PK, efficacy or safety [35]. Any test that is used for patient management decisions should be performed in a certified clinical laboratory. In USA the certification must be compliant with Clinical Laboratory Improvement Amendments (CLIA) to ensure that the clinical laboratory operates under standards for overall quality management system in order to ensure best clinical laboratory practices. If the validated biomarker test will be performed in different CLIA-certified laboratory for a clinical trial, analytical validation and CLIA requirements for the same test should be met by each laboratory, working with the primary laboratory [36].

Today, genetic markers (like SNPs) in LD with validated variants in genes involved in metabolism of specific drugs could be optimally translated into clinical practice through the design of a custom tests. Moreover, a more comprehensive interpretation of knowledge on factors influencing the correlation genotype/phenotype, could improve test diffusion toward the choice of therapeutic options or strategies in presence of a known polymorphic variant for a specific drug. An integrative approach could facilitate the pre-emptive diffusion of biomarker test in clinical practice. Another aspect to consider is the opportunity, by available PGx tools, to discover new genomic variants involved in non-predictable variability in patient drugs response. In fact, NGS-based PGx assays, can sequence all known genetic variants in the human genome and also agnostically discover unknown variants but with the risk of false positive or misidentification. NGS-based PGx platforms applied to study variability in drug response and ADRs indeed contributed to the identification of rare genetic variants, not previously discovered or reported, influencing the phenotype more than common variants traditionally tested for. Indirect influence of environmental or other genetic factors as sex, co-morbid conditions, poly-therapies and ethnic factors on phenotype might conditioned treatment failure [37]. Molecular signatures and integrated PGx information elaborated by efficient and validated algorithms could increase diagnostic accuracy in the population. Until now, available information can be used for recommendations on avoidance or dose reductions: the hope in next future of implementation in daily practice in a more directed and evidence-based fashion in order to lead to an improvement of patient outcome. 

## 6. Promise and Challenges of Pharmacogenomics Fallout in Clinical Practice

Technologic advances in PGx discovery have accelerated the translation of new biomarkers in clinical practice for patient’s stratification according to the genetic background, in order to achieve better treatment outcomes and safety. In the era of precision medicine, until now, many patients do not respond appropriately to therapy or might not be treated fittingly or might suffer delays in therapy shift or withdrawal because of lack of drug efficacy or ADRs. The unpredictable patient’s response to a given therapeutic regimen till represents a significant safety risk. The possibility to prevent such reactions by PGx derived tools is a good chance in terms of lowering health care costs, improve disease management and patient’s outcome. Biomarker testing may also be useful for predicting drug interactions during poly-therapies, and 20–25% of drugs are in part metabolized by cytochrome P450 2D6 (CYP2D6). *CYP2D6* is a highly polymorphic phase 1 gene, mostly non-inducible, but which can be inhibited by many drugs given simultaneously, resulting in poor, intermediate, efficient or ultra-rapid metabolizer (UMs) patient phenotype which condition PK of its substrate drugs [30,38,39]. The testing of tumor tissue bio-markers in cancer clinical care decision making have changed the paradigm of cancer treatment of and they are mandatory in several treatment guidelines for precision oncology [40,41,42,43]. No similar landscape can be presently depicted for PGx biomarkers.

Although a currently available tool for precision oncology is the opportunity to consider the homozygosity for allelic variant UGT1A1*28 before giving irinotecan, taking into account that recommendation to test is already included in the drug sheet [44], it is hard to consider this assay as common clinical practice. Although, guideline-recommended PGx tests for specific tumors are available and required for clinical decision making, as in the case of UGT1A1*28, often the timing in the execution and interpretation of the tests is not optimal and the costs are elevated. On the other hand, the acceptability of the tests is high and the impact on patient consideration has been clearly demonstrated. One important barrier for introducing PGx into clinical practice and during early phase clinical trials is the failure to validate genetic biomarker associations with drug safety or efficacy in independent patients’ cohorts as previously discussed. The validation lack is also due to inadequate sample size for overestimation of the effect [45], the impact of ethnicity that influence the replication of PGx research [46] the drug-drug interactions. It can be concluded that the optimal diffusion of PGx testing in the clinical practice is still hampered by poor availability of validated biomarker analysis and by the lack of sufficient inter-institutional effort for exploiting the whole discovery-validation path. This can partially be due to limited interest by corporate stakeholders while academic research is often hampered by lack of public or charity funding.

## 7. Conclusions

The complex scenario underlying drug efficacy and toxicity highlights the need for a personalized prescription, taking into account phenotype, environmental factors, and genetic background in order to improve treatment benefit and reduce ADRs. PGx studies have allowed the identification of biomarkers for diagnostic or predictive purposes; much of them are, at the present, in the validation phase for clinical translation. Germline genotyping will contribute to highlight molecular mechanisms underlying interactions between SNP linked variants in ADME genes and xenobiotics metabolism, offering a new tool for studies on cancer susceptibility through discovery of complex pathways of interaction. The integration of all PGx information available in validated database will represent the way to design harmonized guidelines that will contribute to implementation efforts of PGx programs in clinical practice. The present limits in fully exploiting the PGx resources clearly indicates the need of coordinated efforts within all the stakeholders in this challenging area of investigation and clinic translation.

## Figures and Tables

**Table 1:** Comparison between pharmacogenomics approaches.

PGx Approach	GWAS	SNPs Panel	Candidate SNP
**Sample size**	Tailored for large populations	Tailored for small populations	Tailored for small populations
**Number of investigated markers**	Larger numbers	1–2 thousand	Smaller number
**Hypothesis**	Hypothesis-free and hypothesis generating	Hypothesis-free and hypothesis generating/PK and PD coverage	Selected on a priori knowledge
**Study Design**	Exploratory	Confirmatory/Exploratory	Confirmatory
**Limitations**	False Negative/control for multiple testing	Coverage of limited genes	False positive/non-replication of results/low genetic coverage

PGx: pharmacogenomics; GWAS: genome-wide association study; SNP: single nucleotide polymorphism.

**Table 2:** PGx discovery platforms.

Platform	TaqMan Open Array PGx Express Panel (Thermo Fisher Scientific)	DMET Plus (Thermo Fisher Scientific)	PharmacoScan (Thermo Fisher Scientific)	Ion AmpliSeqPGx (Thermo Fisher Scientific)	iPLEX ADME PGx (Sequenom)
**Markers (SNP/indels/CNV)**	60	1936	4627	141	192
**Genes**	14	231	1191	40	38
**Sample per assay**	46	48	22, 94	48	3, 12, 48
**DNA input**	10 ng	60 ng	50 ng	10 ng	80 ng
**Technology**	Real-Time PCR	Microarray	Microarray	Next-generation sequencing	Mass spectrometry
**Turnaround time**	~1 day	~3 days	~5 days	~1.5 days	~8 h
**Average Call Rate**	>99.8%	>99.8%	>99.0%	99.8%	>99.0%
**Concordance to reference**	≥99.5%	≥99.5%	≥99.5%	99.9%	98.9%
**Reproducibility**	≥99.8%	≥99.8%	≥99.8%	99.7%	>99.7%
**For research only**	Yes	Yes	Yes	Yes	Yes

SNP: single nucleotide polymorphism, CNV: Copy Number Variation.
